# Nitrogen sources differentially affect respiration, growth, and carbon allocation in Andean and Lowland ecotypes of *Chenopodium quinoa* Willd

**DOI:** 10.3389/fpls.2023.1070472

**Published:** 2023-06-20

**Authors:** María Paz Jerez, José Ortiz, Catalina Castro, Elizabeth Escobar, Carolina Sanhueza, Néstor Fernández Del-Saz, Miquel Ribas-Carbo, Teodoro Coba de la Peña, Enrique Ostria-Gallardo, Susana Fischer, Patricio Alejandro Castro, Luisa Bascunan-Godoy

**Affiliations:** ^1^ Laboratorio de Fisiología Vegetal, Departamento de Botánica, Facultad de Ciencias Naturales y Oceanográficas, Universidad de Concepción, Concepción, Chile; ^2^ Grup de Recerca en Biologia de les Plantes en Condicions Mediterranies, Universitat de les Illes Balears, Carretera de Valldemossa, Palma de Mallorca, Spain; ^3^ Laboratorio de Fisiología Vegetal, Centro de Estudios Avanzados en Zonas Áridas (CEAZA), La Serena, Chile; ^4^ Laboratorio de Fisiología Vegetal, Departamento de Producción vegetal Facultad de Agronomía, Universidad de Concepción, Concepción, Chile; ^5^ Departamento de Fisiología, Facultad de Ciencias Biológicas, Universidad de Concepción, Concepción, Chile

**Keywords:** ammonium, nitrate, ammonium-toxicity, landraces, photosynthetic performance, C metabolism, oxygen-isotope fractionation, alternative- oxidase

## Abstract

*Chenopodium quinoa* Willd. is a native species that originated in the High Andes plateau (Altiplano) and its cultivation spread out to the south of Chile. Because of the different edaphoclimatic characteristics of both regions, soils from Altiplano accumulated higher levels of nitrate ( ${\text{NO}}_{3}^{-}$ ) than in the south of Chile, where soils favor ammonium (NH_4_^+^) accumulation. To elucidate whether *C. quinoa* ecotypes differ in several physiological and biochemical parameters related to their capacity to assimilate ${\text{NO}}_{3}^{-}$ and NH_4_^+^, juvenile plants of Socaire (from Altiplano) and Faro (from Lowland/South of Chile) were grown under different sources of N ( ${\text{NO}}_{3}^{-}$ or NH_4_^+^). Measurements of photosynthesis and foliar oxygen-isotope fractionation were carried out, together with biochemical analyses, as proxies for the analysis of plant performance or sensitivity to NH_4_^+^. Overall, while NH_4_^+^ reduced the growth of Socaire, it induced higher biomass productivity and increased protein synthesis, oxygen consumption, and cytochrome oxidase activity in Faro. We discussed that ATP yield from respiration in Faro could promote protein production from assimilated NH_4_^+^ to benefit its growth. The characterization of this differential sensitivity of both quinoa ecotypes for NH_4_^+^ contributes to a better understanding of nutritional aspects driving plant primary productivity.

## Introduction

Carbon (C) assimilation and release in plants are key plant biochemical processes for global primary productivity. It is dependent on multiple processes ranging from photosynthesis, which absorbs energy from sunlight to build carbohydrates, to aerobic cellular respiration, which releases metabolic energy to be captured by the cell in the form of ATP during mitochondrial oxidative phosphorylation (Del-Saz et al., 2018; O’Leary et al., 2019). Both photosynthesis and respiration and, thus, plant growth are dependent on nitrogen (N) plant status (Miller and Cramer, 2005). This essential macronutrient is mostly uptaken from soils by roots in different inorganic forms (Miller and Cramer, 2005; Kant et al., 2011; Xu et al., 2012). Nitrate ( ${\text{NO}}_{3}^{-}$ ) can be a dominant form of N in arid and semi-arid regions with basic and aerated soils, where alkali compounds are accumulated. Conversely, NH_4_^+^ is a common N source in regions with high precipitations and acidic soils, where soluble basic molecules including ${\text{NO}}_{3}^{-}$ are leached (Luzio et al., 2002; Blake, 2005; Miller and Cramer, 2005; Hachiya and Sakakibara, 2017). Several studies suggest that climate variations could change the ratios of the inorganic forms of N ( ${\text{NO}}_{3}^{-}$ and NH_4_^+^) available for plants (Myers et al., 2014; Coskun et al., 2016; Greaver et al., 2016; Schittek et al., 2016; Ackerman et al., 2019). Climate change may potentially increase NH_4_^+^ over ${\text{NO}}_{3}^{-}$ by slowing down the rate of nitrification (the aerobic oxidation of NH_4_^+^ to ${\text{NO}}_{3}^{-}$ ) (Auyeung et al., 2015). Precisely, the decrease in rainfall, together with an increase in temperature and a decrease of pH in soils, could compromise biological nitrification rather than denitrification, altering the availability of inorganic forms of N in soils (Breuer et al., 2002; Barnard et al., 2006; Auyeung et al., 2015; Coskun et al., 2016; Greaver et al., 2016; Daryanto et al., 2019).

Compared to ${\text{NO}}_{3}^{-}$ , NH_4_^+^ assimilation entails a lower energy cost, and can be beneficial for plant growth in many circumstances, including elevated levels of CO_2_ (Rubio-Asensio and Bloom, 2017). However, some plants are sensitive to NH_4_^+^ due to its toxicity, displaying growth suppression and chlorosis (Rubio-Asensio et al., 2015). Alterations in photosynthesis and PSII performance have been described in plants growing under NH_4_^+^ because of increased synthesis of reactive oxygen species (ROS) (Zhu et al., 2000; Guo et al., 2005; Alencar et al., 2019). Regarding respiration, several studies have shown increases in O_2_ consumption in plants growing under ${\text{NH}}_{4}^{+}$ as the sole N source (Rigano et al., 1996; Britto et al., 2001; Escobar et al., 2006). It was suggested that exposure to NH_4_^+^ could alter respiratory activity in leaves for the benefit of redox homeostasis through the modulation of the activities of the cytochrome oxidase pathway (COP) and the non-phosphorylating alternative oxidase pathway (AOP; Rigano et al., 1996; Britto et al., 2001; Escobar et al., 2006; Ortiz et al., 2020), which can be measured *in vivo* by mass spectrometry (Del-Saz et al., 2018). However, no previous studies have characterized the respiratory activities of COP and AOP in plants grown under different N sources.

*Chenopodium quinoa* Willd. (Amaranthaceae family) is an important crop for food security worldwide (FAO, 2011). It has been reported that quinoa was originated in the Altiplano of the Andes (3500 m.a.s.l., shared by Peru, Bolivia, and Chile) and spread out to Southern Chile by the Inca Empire (Martinez et al., 2009). However, it has recently been suggested that highland and lowland/coastal plants were domesticated independently in these environments (Planella et al., 2015; Jarvis et al., 2017; Maughan et al., 2019). In the Altiplano, quinoa plants deal with extreme drought, high-flux solar radiation, very strong daytime temperature changes, limited volume of annual rainfall (150–300 mm/year), and saline-alkali soils (García et al., 2007; Cárdenas-Castillo et al., 2021; Rascón et al., 2021). In the South of Chile, quinoa plants face acidic soils, uneven N content in the soil, and rainfall ranging from 500 to 1,500 mm/year (Luzio et al., 2010; Bascuñán-Godoy et al., 2018). Previous studies described that ${\text{NO}}_{3}^{-}$ availability is an important factor that helps determine differences between these ecotypes at both biochemical and metabolic levels (Pinto-Irish et al., 2020). These authors found that the Andean ecotype displayed a more efficient mechanism of ${\text{NO}}_{3}^{-}$ uptake than the southern ecotype, displaying a higher level of proteins in leaves and roots. Additionally, seeds presented higher levels of amino acids, and metabolites from shikimate, ornithine, purine, and nicotinamide metabolism. It remains to be determined whether the N source is a factor that may lead to different plant physiological performance. It has been described that alternative respiration plays different roles under abiotic stress conditions (Del-Saz et al., 2018) depending on plant species or genotypes (Florez-Sarasa et al., 2016; Del-Saz et al., 2018; Del-Saz et al., 2021). Bearing in mind that Chile is one of the most climatically diverse places on the planet, there is a need to characterize respiration metabolism under different scenarios of N forms available for uptake. In this sense, quinoa is an optimal species because the distribution of this species is geographically wide in Chile and diverse ecotypes have been described (Bazile et al., 2014).

In the present research, we categorized two places in Chile with a different predominance of ${\text{NO}}_{3}^{-}$ or NH_4_^+^ forms (Luzio et al., 2010) according to the existence of a longitudinal gradient of pH from the North (alkaline) to the South (acidic) of this country (SoilGrids, 2021). We performed measurements of photosynthesis, oxygen-isotope fractionation, total soluble sugars, starch, NH_4_^+^, protein, chlorophylls, and betacyanin contents in two ecotypes of quinoa plants from Altiplano (Socaire) and South of Chile (Faro) grown under different N sources as proxies for the evaluation of plant performance or sensitivity to NH_4_^+^. Thus, our main objective was to characterize plant respiratory parameters in leaves of both *C. quinoa* ecotypes grown under ${\text{NO}}_{3}^{-}$ and NH_4_^+^. Secondly, we discuss possible respiratory differences based on other biochemical parameters related to plant performance and N assimilation that help to provide first insights into the regulation of the respiratory pathways by this nutrient.

## Materials and methods

### Plant material

Seeds of two Chilean ecotypes of *C. quinoa* (Willd.) from contrasting agro-ecological origins were used in the experiments: Socaire from the Chilean Altiplano (Socaire, 23°35’31.58” S, 67°53’17.69” W, and 3,500 m.a.s.l.) and the Lowland/Southern coastal Faro ecotype (Chillan, 36°35’ 43.2” S, 72°04’ 39.9” W and 140 m.a.s.l.). Seeds of Socaire were collected in a private field (23°34′S 67°54′W, 3,500 m.a.s.l.). The soil in the zone is basic (pH between 7.8 and 8.8) and the total percent and available N were 0.09% and 46 mg/kg, respectively. Soil and climate characterizations of the zone can be found in García et al. (2007). Faro seeds were collected in Chillan at “El Nogal Experimental Station” (36°35’ 43.2” S, 72°04’ 39.9” W and 140 m.a.s.l.). The pH of the soil was approximately 4.5, and the total percent and available N were 0.01% and 44 mg/kg, respectively. Soil and climate characterizations in this station are found in Fischer et al. (2013) and Stolpe (2006). Seeds of both ecotypes (Socaire and Faro) were collected in summer (February) in each location (in Socaire soils and in Chillan soils, respectively). Seeds of both ecotypes are included in the National Seed Bank of Chile managed by the Genetic Resources section of the National Institute of Agriculture Research (http://163.247.128.32/gringlobal/search.aspx, INIA-Intihuasi Vicuña, Chile).

### Determination of optimal N supply

In order to compare the performance of both ecotypes under ${\text{NO}}_{3}^{-}$ and NH_4_^+^, we established an optimal N concentration for plant growth. We set up biomass curves under the supply of different amounts of N, using ${\text{NO}}_{3}^{-}$ as a referential condition because it is generally the preferred N source for plants without toxic effects. Germinated seeds of both ecotypes with similar lengths of emerged radicle were transplanted into 700-ml pots containing sand:perlite (1:1), and six concentrations of ${\text{NO}}_{3}^{-}$ (using KNO_3_ salt) and NH_4_^+^ [using (NH_4_)_2_SO_4_ salt] were tested: 0.0, 5.0, 10, 20, 40, and 100 mM. Ten pots (with three plants each) were submitted to each N concentration of ${\text{NO}}_{3}^{-}$ or NH_4_^+^. The conditions of the growing chamber and the irrigation solution were the same as explained below. Thirty-day-old plants were collected for total biomass determination.

### Plant growing conditions

Socaire and Faro plants reached maximum biomass at 20 mM of N (**Supplementary Figure 1**). Therefore, this amount was used for the physiological experiments comparing N sources. Plants were germinated and grown in pots supplied with 20 mM of ${\text{NO}}_{3}^{-}$ or NH_4_^+^ using the following growing conditions: light intensity of 575 µmol/m^2^/s, 21°C/19°C day/night, 16 h light/8 h dark photoperiod, and 75% relative humidity. Plants were supplied once with MS 407 nutrient medium described by Murashige and Skoog (1962), and consisting of the following: 0.30 mM MgSO_4_.7H_2_O, 0.22 mM CaCl_2_, 0.62 mM KH_2_PO_4_, 12.7 mM KCl, 0.05 µM KI, 1.00 µM H_3_BO_3_, 1.32 µM MnSO_4_.4H_2_O, 0.30 µM ZnSO_4_.7H_2_O, 0.01 µM Na_2_MoO_4_.2H_2_O, 0.001 µM CuSO_4_.5H_2_O, 0.001 µM CoCl_2_.6H_2_O, 0.51 µM Na_2_.EDTA, 0.50 µM FeSO_4_.7H_2_O, 2.78 µM inositol, 0.02 µM nicotinic acid, 0.01 µM pyridoxine HCl, 0.001 µM thiamine-HCl, and 0.13 µM Glycine. KNO_3_ and (NH_4_)_2_SO_4_ varied according to the treatment. The pH was set at 5.8.

Thirty pots of 700 ml (three plants per pot) were used for each N source and ecotype. The experiment was run in a completely randomized design and additional plants were grown to prevent the bordering effect. Juvenile plants were collected 30 days after sowing at midday. Harvested plants for biochemical analysis were fractionated into belowground and aboveground (leaves and biomass). The root samples were rinsed in distilled water and dried with a paper towel. The tissue samples were frozen in liquid N and then freeze-dried. Tissue was ground to a fine powder in liquid N using a mortar and pestle and stored in Falcon tubes at −80°C. Measurements were performed belowground and aboveground, except for pigments that were performed in leaves.

### Plant growth analyses

Images of plants and leaves submitted to the different treatments were taken with the Scanner Epson Perfection V850 Pro Photo (Epson Corporation, San Jose, CA). The image acquisition parameter was set to “high” accuracy (600 dpi; image size 18 MB). Leaf area was measured through image analysis using the ImageJ software (NIMH, Bethesda, Maryland, USA). Above and belowground biomasses were determined by drying the tissues at 60°C for 48 h till constant weight.

### Determination of the percentage of C and N per dry matter

N and C elements were measured in the whole plant from the different N treatments and ecotypes (*n* = 3). Briefly, plant tissue was oven-dried at 60°C for 48 h and ground. A subsample of 5 mg was weighed and stored in plastic vials. Samples were measured using the Elemental Combustion System CHNS-O (Costech Analytical Technologies Inc., Valentia, USA). C and N are reported as the percentage of elements per dry matter.

### Determination of total soluble sugars, starch, and NSCs

We used above- and belowground tissue from each ecotype and N source (*n* = 6). Changes in total soluble sugars (TSS), starch, and total nonstructural carbon (NSC) in aerial and roots were assayed following the method of Marquis et al. (1997). Total soluble sugars were separated and extracted with methanol/chloroform/water according to and determined by colorimetry using 2% phenol and sulfuric acid at 490 nm according to Dickinson (1979) and Chow et al (2004). The insoluble fraction that contains starch was hydrolyzed to glucose overnight using a sodium acetate buffer and amyloglucosidase (Sigma-Aldrich 10115, St. Louis, MO, USA) at 45°C and then measured with a phenol–sulfuric acid reaction as in Marquis et al. (1997).

### NH_4_^+^ and protein quantification

Total soluble protein and ammonium contents were determined in the above- and belowground tissue of the two quinoa ecotypes studied. NH_4_^+^ was determined according to Forster (1995). Absorbance was measured at 660 nm in a spectrophotometer (Infinite 200 Pro, Tecan, Männedorf, Switzerland). We used the TCA/acetone procedure for protein extraction (Wang et al., 2008). The Quick Start Bradford Assay kit (Bradford, 1976) was used for protein quantification using BSA as the standard protein (*n* = 4), according to manufacturer instructions (Bio-Rad, Hercules, CA, USA).

### Chlorophylls and betacyanins in leaves

Chlorophylls *a* and *b* were extracted from leaves of plants of all treatments (*n* = 6) in 80% of acetone overnight and centrifuged at 12,000*g for* 10 *min*. The content of chlorophylls was determined at 664 and 647 nm following the method described by Lichtenthaler and Buschmann (2001).

Betacyanins were extracted from leaves (*n* = 6) in water/methanol and spectrophotometrically determined at 536 nm. The betacyanin content of the plant aqueous extracts was estimated according to Abderrahim et al. (2015).

### Lipid peroxidation

The lipid peroxidation in the above- and belowground tissue of the two quinoa ecotypes studied (*n* = 5) was determined *in vitro* by estimating the formation of malondialdehyde (MDA) according to the method described by Ortega-Villasante et al. (2005). Frozen leaf tissue (0.1–0.2 g) was homogenized in 1 ml of TCA–TBA–HCl reagent [15% (w/v)] trichloroacetic acid, 0.37% (w/v) 2-thiobarbituric acid, 0.25 M HCl, and 0.01% butylated hydroxytoluene. After homogenization, samples were incubated at 90°C for 30 min and centrifuged at 12,000 *g* for 10 min. Absorbance was measured at 535 nm and 600 nm.

### Gas exchange

Gas exchange measurements of net photosynthesis (A_N_) were performed in leaves from both ecotypes and N sources in six plants per treatment, using a portable photosynthesis system (Li-6400XT, LI-COR Inc., Lincoln, NE, USA) equipped with a light source (6200-02B LED, Li-Cor).

Light curves were run to determine the light saturation intensity at which plants reached maximum photosynthesis. A_N_ rates were measured at mid-morning (between 11 a.m. and 1 p.m.) with the gas exchange previously stabilized. Conditions in the leaf chamber were as follows: block temperature of 25°C, 1,500 µmol photon m^−2^ s^−1^, an air flow of 300 (mol s^−1^), and a CO_2_ concentration (Ca) of 400 mol mol^−1^. Relative humidity ranged between 45% and 50% and VPD ranged between 1.6 and 1.9 kPa. A_N_ data were normalized by the area of leaves (*n* = 6).

### Respiration and oxygen-isotope fractionation measurements

For respiratory measurements, leaves of quinoa plants, which were grown at the University of the Balearic Islands under similar growing conditions, were placed in a 3-ml stainless-steel closed cuvette and maintained at a constant temperature of 25°C. Air samples were sequentially removed from the cuvette and fed into the mass spectrometer (Delta XPlus; Thermo LCC, Bremen, Germany). Changes in the ^18^O/^16^O ratios and O_2_ concentration were obtained to calculate the oxygen-isotope fractionation and the electron partitioning to the AOP (τ_a_), allowing calculations of the *in vivo* activities of AOP and COP as described in Del-Saz et al. (2017). End point fractionation values of the AOP (Δ_a_) were determined in leaves with a solution of 25 mM potassium cyanide (KCN) for 20 min. A value of 32.8 ± 0.69‰ (*n* = 3) was obtained in leaves of the Faro ecotype. Owing to a limitation in the number of Socaire plants, we assumed a similar value of Δ_c_ of 32.8‰. We also assumed a value of 20.0‰ for the endpoint fractionation values of the COP (Δ_c_) as this has been shown to be constant in most leaves and species examined (Ribas-Carbo et al., 2005). Values presented are the mean of one measurement in four plants per ecotype that were performed from 9 a.m. to 5 p.m. during five consecutive days.

### Statistical analysis

For the determination of sufficient N source supply, we used three-way ANOVA (level of significance *p* < 0.05) using ecotype, source of N, and concentration as factors. Data from the effects of N source ( ${\text{NO}}_{3}^{-}$ or NH_4_^+^) on the different quinoa ecotypes (Socaire and Faro) were analyzed by two-way ANOVA. Tukey HSD test was used to identify means with significant differences (level of significance *p* < 0.05).

## Results

### Growing under different N sources

The total dry biomass of both ecotypes enhanced with increased N supply, until reaching mean maximum biomass values at 20 mM N (**Supplementary Figure 1**). Consistently with previous results published by Pinto-Irish et al. (2020), we considered 20 mM N as optimal for growing conditions (because both ecotypes reached the highest biomass either under ${\text{NO}}_{3}^{-}$ or NH_4_^+^). At higher concentrations (40 and 100 mM), plant biomass decreased under ${\text{NO}}_{3}^{-}$ , and mortality increased under NH_4_^+^; for this reason, these data were omitted in **Supplementary Figure 1**. Considering that ${\text{NO}}_{3}^{-}$ is generally the preferred N source without deleterious effects, our results focused on the comparison of the ecotypes under NH_4_^+^ regarding their performance under ${\text{NO}}_{3}^{-}$ .

**Figure 1:**
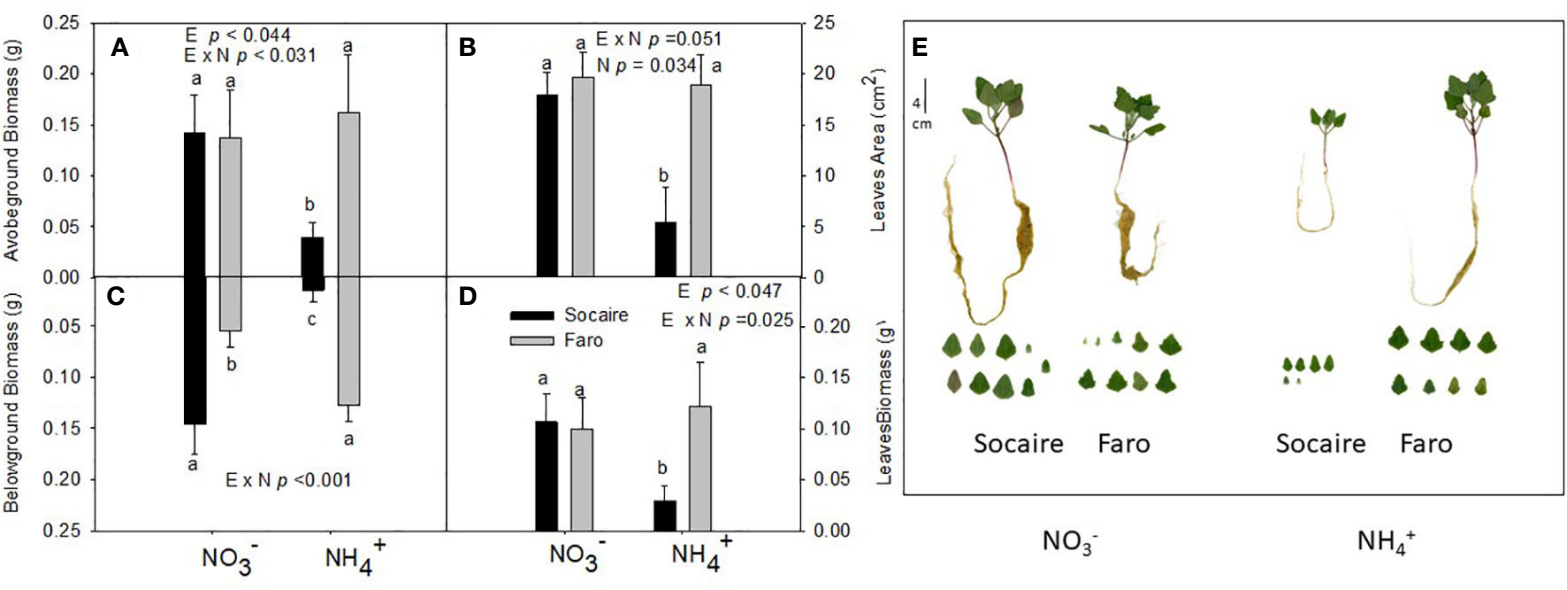
Biometrical parameters: aboveground biomass **(A)**, total leaf area **(B)**, belowground biomass **(C)**, total leaves biomass **(D)**, and representative images of whole plants and leaves **(E)** under different N sources in two ecotypes of *C. quinoa*. Plants were subjected to 20 mM ${\text{NO}}_{3}^{-}$ or NH_4_^+^ supply per 30 days. Values are means ± SE (*n* = 4). Different letters show statistical differences using two-way ANOVA considering ecotypes and source of N as factors. Tukey HSD was used as a *post-hoc* test (*p* < 0.05).

The two-way ANOVA reveals interactions between ecotypes (E) and nitrogen (N) source (E × N) in aboveground, belowground, and total leaves biomass (**Figure 1** and **Table 1**). Ecotypes presented similar aboveground biomass under ${\text{NO}}_{3}^{-}$ (**Figures 1A, E**); however, under NH_4_^+^ supply, Socaire showed a 72% reduction in plant biomass, while Faro maintained similar values to those observed under ${\text{NO}}_{3}^{-}$ (*p* < 0.031) (**Figures 1A, E**). Regarding belowground biomass, ecotypes exhibited a large contrasting response under NH_4_^+^ (E × N), with a 80% reduction in Socaire under NH_4_^+^ compared to ${\text{NO}}_{3}^{-}$ , while a twofold increase (*p* < 0.001) was observed in Faro (**Figure 1C**). Both the total leaf area and total leaf biomass per plant (sum of all leaves) (**Figures 1B, D, E**) correlated well with aboveground biomass. No differences in leaf area or leaf biomass were observed between N sources in Faro.

**Table 1:** *p*-values (*p* < 0.05) and the size effects (*η* ^2^; Eta squared) for the effects of E, N, and their interaction determined by two-way ANOVA analysis on biometrics and physiological attributes: above- and belowground biomass, total leaves biomass, total leaves area, %C, %N, C:N ratio, NH_4_^+^, protein, TSS, starch, NSC, and MDA at both above- and belowground, Chlorophyll *a* and *b*, betacyanins, A_N_, *V*_total_, τa, *v*_cyt_, and *v*_alt_ in leaves of two genotypes of *Chenopodium quinoa* grown at two sources of nitrogen supplementation for 30 days.

Response variable	*p*			*η* ^2^		
	E	N	E × N	E	N	E × N
Aboveground biomass	0.044	0.162	0.031	**0.166**	0.081	0.191
Belowground biomass	0.579	0.162	<0.001	0.008	0.050	**0.396**
Leaves biomass	0.047	0.179	0.025	0.161	0.074	**0.204**
Leaves area	0.083	0.034	0.051	0.121	**0.181**	0.152
%C	<0.001	<0.001	<0.001	0.086	**0.431**	0.108
%N	<0.001	<0.001	<0.001	**0.432**	0.066	0.121
C:N ratio	<0.001	<0.001	<0.001	0.206	**0.420**	0.013
${\text{NH}}_{4}^{+}$ aboveground	<0.001	0.059	0.995	**0.3533**	0.0855	0.0000
NH_4_^+^ belowground	0.122	0.993	0.005	0.083	0.000	**0.267**
Protein aboveground	<0.001	<0.001	0.001	0.767	**0.857**	0.486
Protein belowground	<0.001	<0.001	<0.001	0.106	0.279	**0.297**
TSS aboveground	0.2	<0.001	0.208	0.024	**0.424**	0.023
TSS belowground	0.003	<0.001	0.007	0.173	**0.254**	0.143
Starch aboveground	<0.001	0.972	0.818	**0.343**	0.000	0.002
Starch belowground	0.011	0.038	<0.001	**0.135**	0.088	0.309
NSC aboveground	0.01	0.034	0.525	**0.220**	0.150	0.014
NSC belowground	0.003	<0.001	0.005	0.174	**0.246**	0.156
Chl *a*	<0.001	<0.001	<0.001	0.200	0.227	**0.251**
Chl *b*	<0.001	<0.001	<0.001	0.198	0.219	**0.249**
Betacyanins	0.558	0.844	<0.001	0.007	0.001	**0.361**
MDA aboveground	0.099	0.073	0.966	0.119	**0.139**	0.000
MDA belowground	0.283	0.026	0.013	0.034	0.144	**0.179**
A_N_ leaves	0.012	0.077	0.198	**0.215**	0.1082	0.0577
*V*_total_	0.17	0.012	0.811	0.085	**0.276**	0.003
τa	<0.001	0.039	0.02	**0.327**	0.100	0.131
*v*_cyt_	0.555	0.005	0.415	0.015	**0.322**	0.028
*v*_alt_	0.009	0.188	0.277	**0.282**	0.072	0.049

The η^2^ values were calculated from the information in the ANOVA table as η^2^ = Treatment sum of square/(treatment sum of square + total sum of squares). The factor with the largest effect size is indicated in bold.

The underlined values show a significant effect (p < 0.05).

### C and N content under different N sources

**Figure 2:**
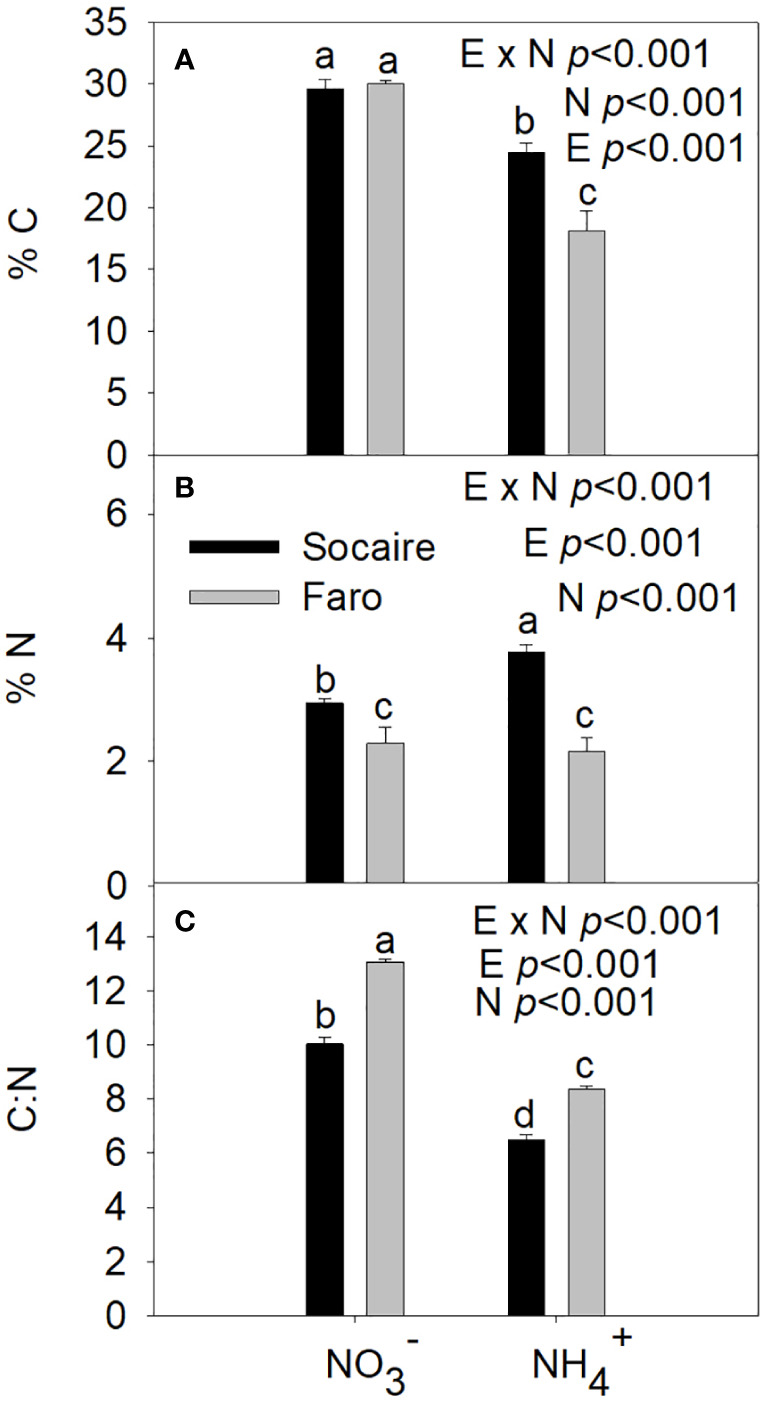
Percent of C **(A)**, N **(B)**, and C:N ratio **(C)** under different N sources ( ${\text{NO}}_{3}^{-}$ and NH_4_^+^) in two ecotypes of *C. quinoa*. Plants were subjected to 20 mM ${\text{NO}}_{3}^{-}$ or NH_4_^+^ supply per 30 days. Values are means ± SE (*n* = 3). Different letters show statistical differences using two-way ANOVA considering ecotypes and source of N as factors. Tukey HSD was used as a *post-hoc* test (*p* < 0.05).

The %C, %N, and the C:N ratio were significantly affected by E × N interaction (**Table 1**). Both ecotypes presented similar %C values under ${\text{NO}}_{3}^{-}$ supply; however, their content was significantly reduced under NH_4_^+^ supply (*p* < 0.001) (**Figure 2A**).

Under both N sources, Socaire displayed an enhanced level of %N compared to Faro (**Figure 2B**). A significant increase in %N was observed in Socaire plants under NH_4_^+^ compared to ${\text{NO}}_{3}^{-}$ conditions. Contrastingly, Faro showed a similar %N under both N sources. Under, the %N in Socaire was twice as high as in Faro.

The C:N ratio was higher in Faro than in Socaire at both conditions. However, both ecotypes showed a significant decrease in the C:N ratio under NH_4_^+^, with the lowest values in Socaire (**Figure 2C**).

### NH_4_^+^ and protein content under different N sources

**Figure 3:**
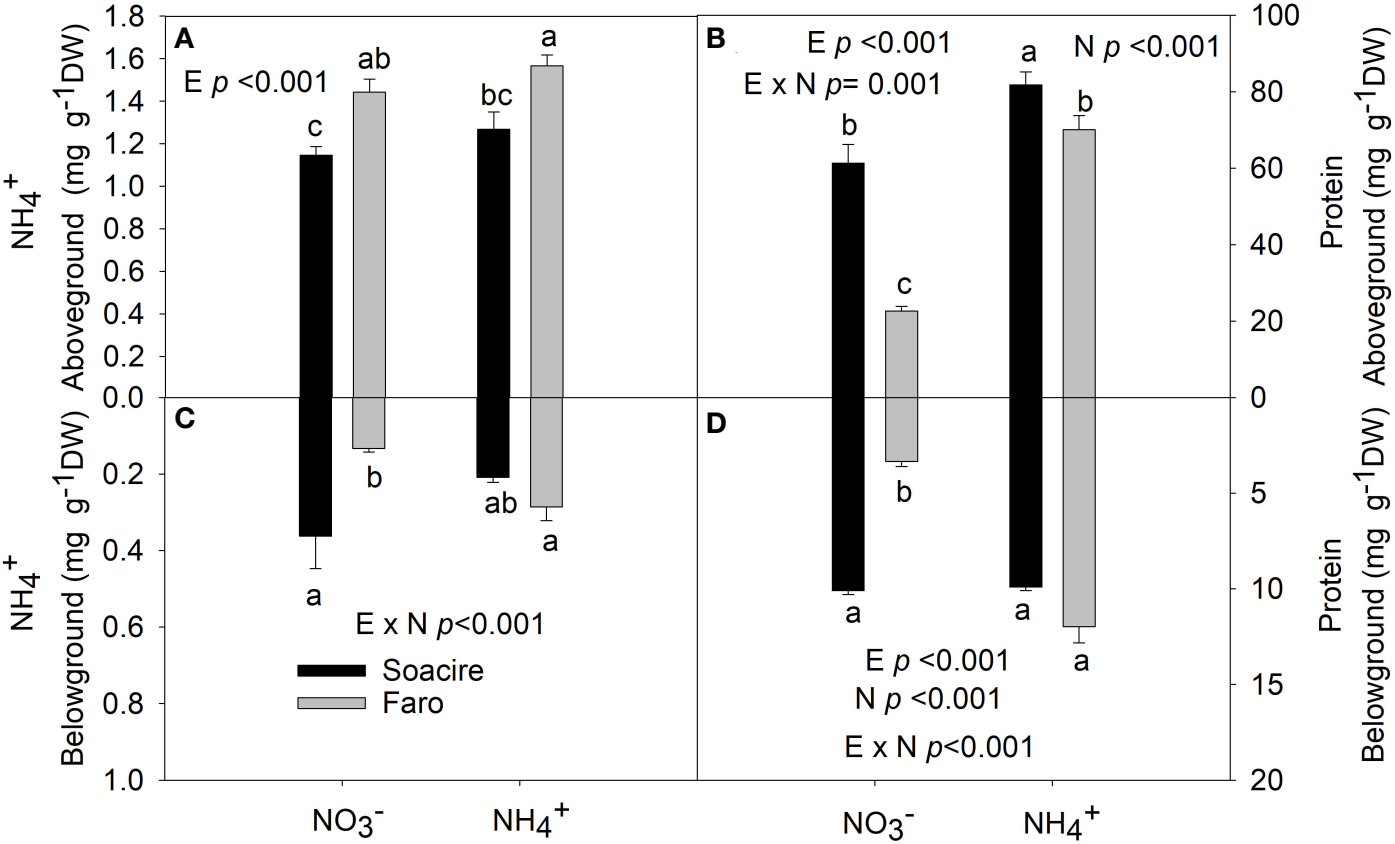
NH_4_^+^ and protein content at aboveground **(A, B)** and belowground **(C, D)** under different N sources in two ecotypes of *C. quinoa*. Plants were subjected to 20 mM NH_4_^+^ or NH_4_^+^ supply per 30 days. Values are means ± SE (*n* = 4). Different letters show statistical differences using two-way ANOVA considering ecotypes and source of N as factors. Tukey HSD was used as a *post-hoc* test (*p* < 0.05).

The two-way ANOVA revealed that NH_4_^+^ content belowground was affected by E × N interaction (*p* < 0.001). The NH_4_^+^ content in roots increased by 30% in Faro ecotype under NH_4_^+^ treatment compared to NH_4_^+^, while it was maintained in Socaire (**Figure 3**). Aboveground NH_4_^+^ was not affected by N sources and differences were based on E (*p* < 0.001).

The two-way ANOVA reveals that protein content at above- and belowground (*p* < 0.001) tissues was affected by E × N interaction (**Table 1** and **Figures 3A, B**). Under ${\text{NO}}_{3}^{-}$ , protein level was three times higher in Socaire than in Faro (in both above- and belowground tissues). Under NH_4_^+^, Faro showed a fourfold increase in protein content in both shoot and root, while Socaire showed an increase of 33% in protein content only aboveground.

### Total soluble sugars, starch, and nonstructural carbon

**Figure 4:**
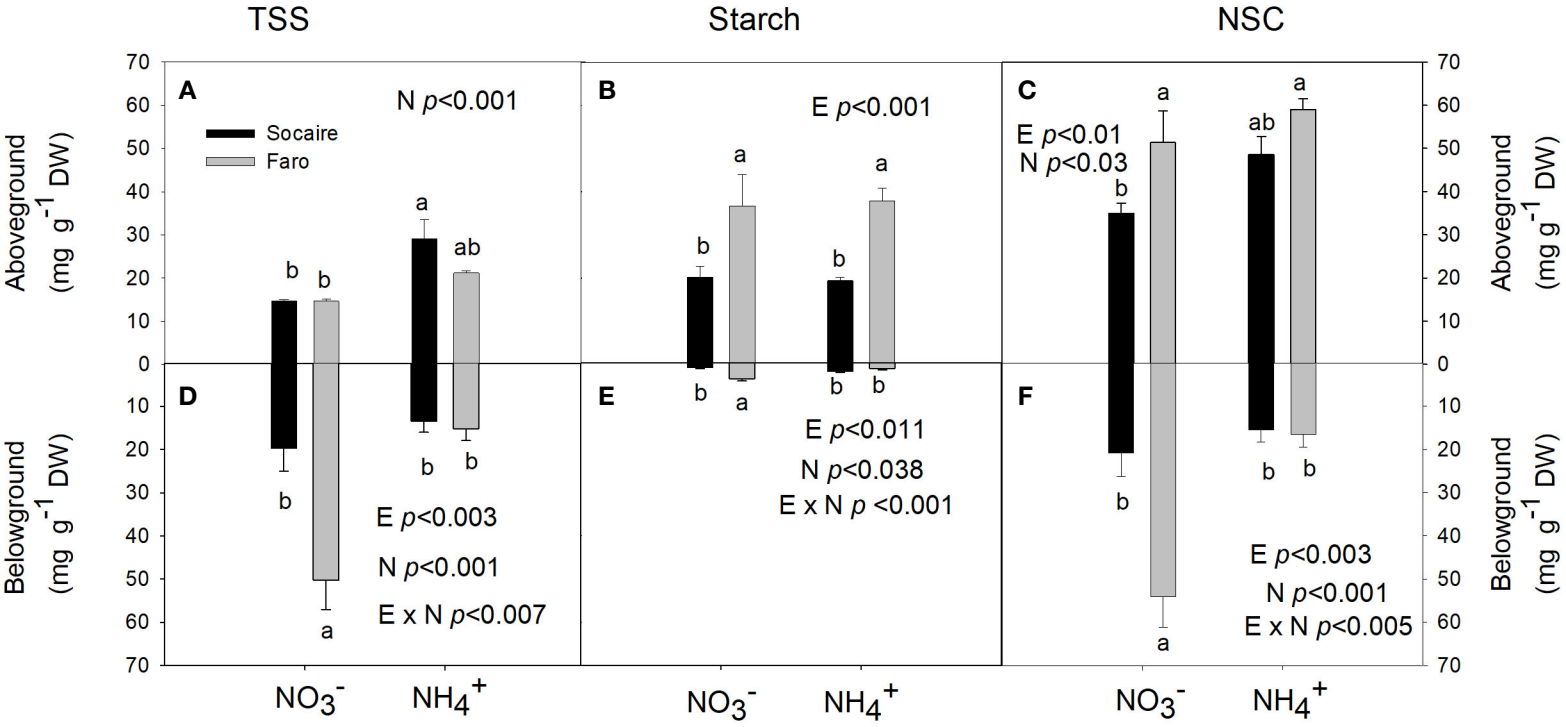
Effect of N source on TSS **(A, D)**, starch **(B, E)**, and NSC **(C, F)** in aboveground and belowground tissues in juvenile plants of two ecotypes of *C. quinoa.* Plants were subjected to 20 mM ${\text{NO}}_{3}^{-}$ or NH_4_^+^ supply per 30 days. Values are means ± SE (*n* = 5). Different letters show statistical differences using two-way ANOVA considering ecotypes and source of N as factors. Tukey HSD was used as a *post-hoc* test (*p* < 0.05).

A non-significant E × N interaction was observed in the storage of carbohydrates aboveground (normalized by dry weight) (*p* > 0.05); however, significant E × N interaction was observed for TSS, starch, and NSC belowground (**Table 1** and **Figure 4**).

In aboveground tissues (stem plus leaves), TSS tended to increase under NH_4_^+^ in both ecotypes and significant differences were observed in Socaire (**Figure 4A**). Starch only depended on E aboveground (*p* < 0.001) (**Figure 3B**). In belowground tissues, Faro showed higher TSS and starch contents than Socaire under ${\text{NO}}_{3}^{-}$ conditions (**Figures 4D, E**). Under ${\text{NO}}_{3}^{-}$ , Faro showed the highest values of NSC in both above- and belowground tissues. However, a significant decrease in belowground NSC was observed under NH_4_^+^, while NSC values in the aboveground tissues reached similar values to those observed in plants under ${\text{NO}}_{3}^{-}$ (**Figures 4C, F**). On the other hand, Socaire displays similar NSC values under both sources of N (**Figure 4**). These results changed drastically when they are interpreted as organs (**Supplementary Figure 2**), where strong reductions were observed in NSC of Socaire under NH_4_^+^ at both aboveground tissues and roots.

### Chlorophylls, betacyanins, and MDA

**Figure 5:**
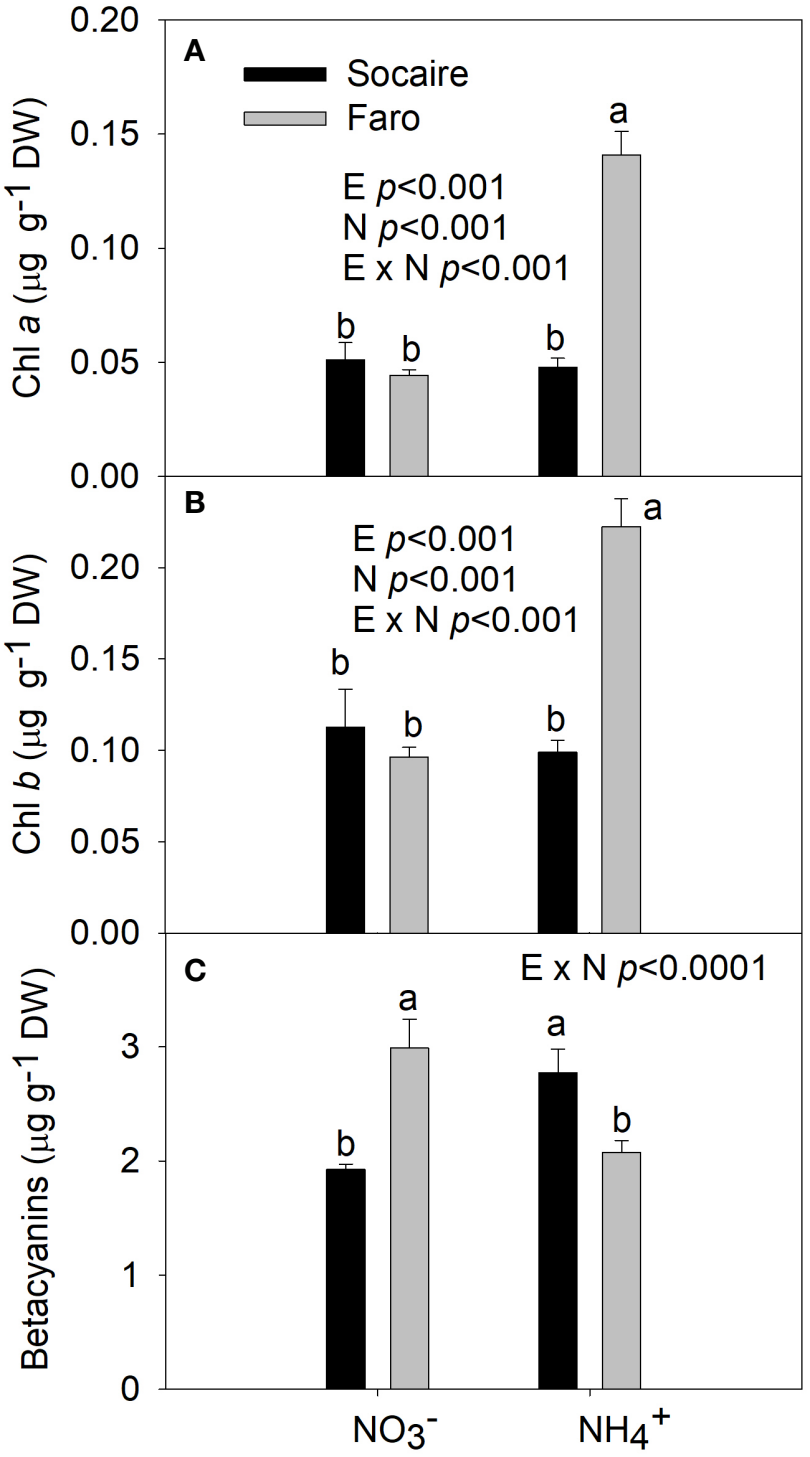
The effect of N sources in Chlorophyll *a* **(A)**, Chlorophyll *b* **(B)**, and Betacyanins **(C)** in leaves of two Quinoa ecotypes. Plants were subjected to 20 mM ${\text{NO}}_{3}^{-}$ or NH_4_^+^ supply per 30 days. Values are means ± SE (*n* = 6). Different letters show statistical differences using two-way ANOVA considering ecotypes and source of N as factors. Tukey HSD was used as a *post-hoc* test (*p* < 0.05).

A significant E × N interaction was observed for chlorophylls, betacyanins, and MDA (*p* < 0.001). Chlorophylls *a* and *b* increased in Faro under NH_4_^+^ compared to ${\text{NO}}_{3}^{-}$ , while no significant changes were observed in Socaire (**Figures 5A, B**). Regarding betacyanins, an increase of 30% was observed in Socaire under NH_4_^+^ compared to NO_3_, displaying significantly higher values than those observed in Faro (**Figure 5C**).

**Figure 6:**
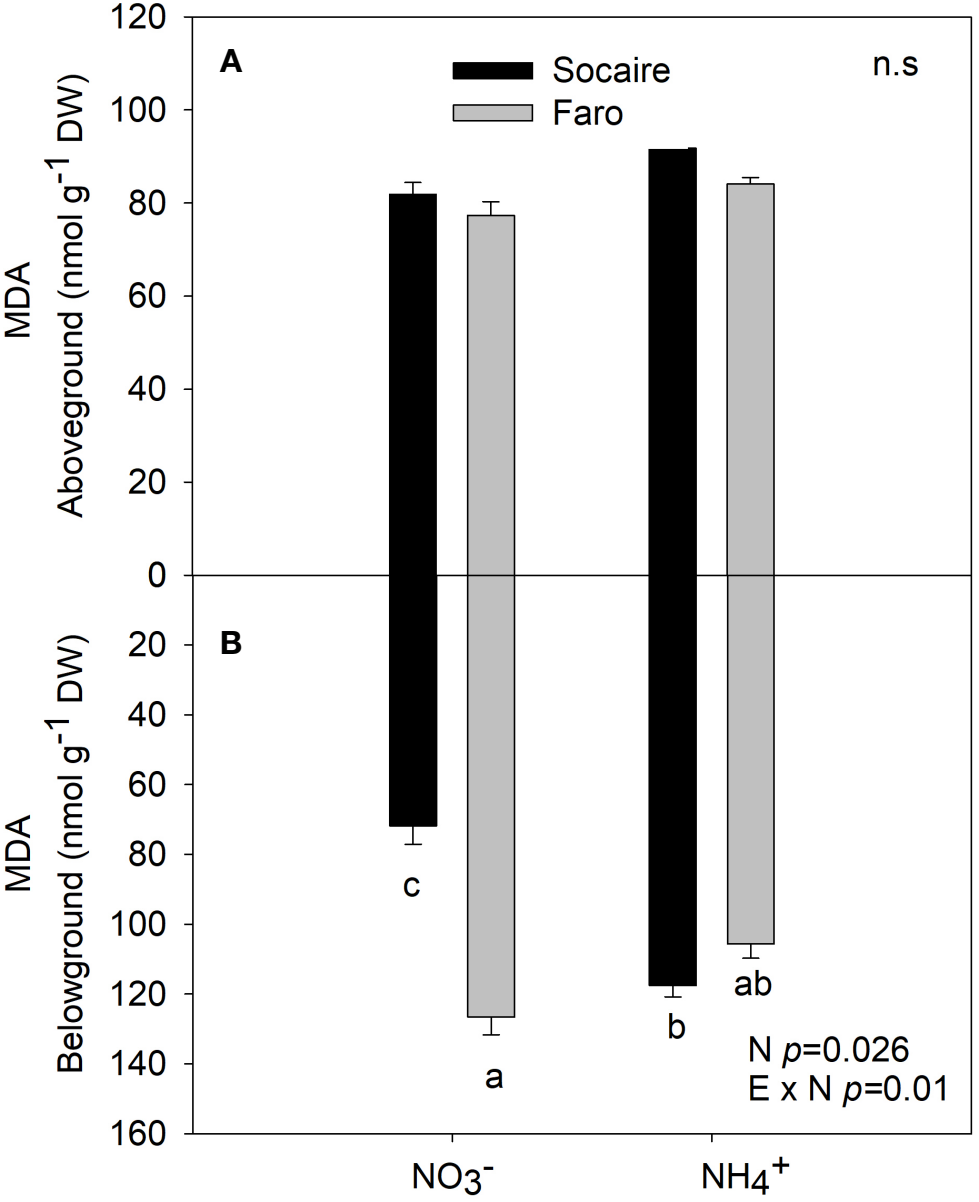
Lipid peroxidation (measured as MDA) at aboveground **(A)** and belowground **(B)** under different N sources in two ecotypes of *C. quinoa*. Plants were subjected to 20 mM ${\text{NO}}_{3}^{-}$ or NH_4_^+^ supply per 30 days. Values are means ± SE (*n* = 5). Different letters show statistical differences using two-way ANOVA considering ecotypes and source of N as factors. Tukey HSD was used as a *post-hoc* test (*p* < 0.05).

An increase of 50% in belowground MDA content was observed in the Socaire ecotype under NH_4_^+^ compared to ${\text{NO}}_{3}^{-}$ (**Figure 6**), while similar values were observed aboveground in comparison to Faro under ${\text{NO}}_{3}^{-}$ and NH_4_^+^. Faro maintained MDA levels under both N sources (**Figure 6D**).

### Gas exchange: photosynthesis

**Figure 7:**
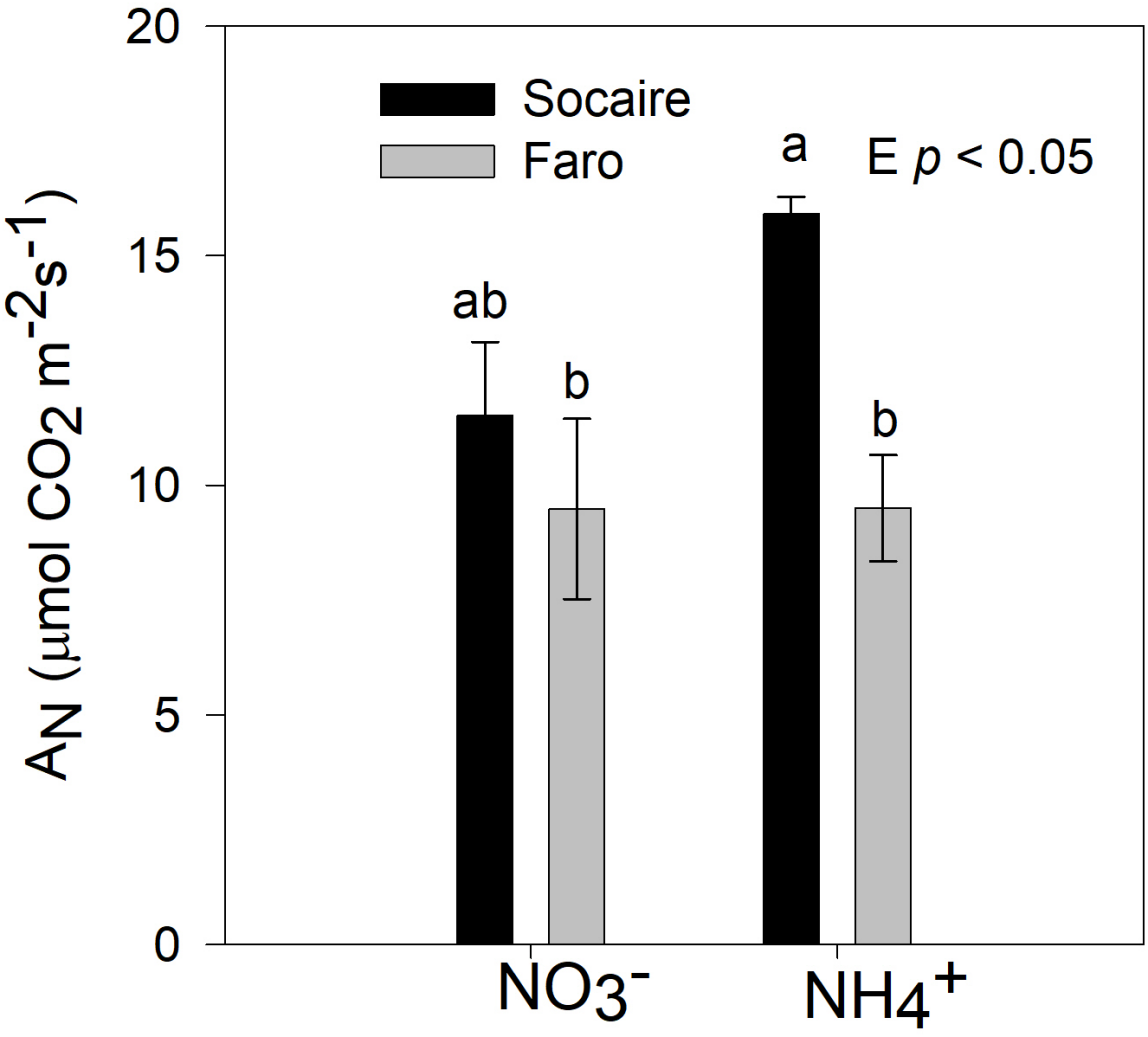
Net photosynthesis rates were taken in well-developed leaves on juvenile plants of two ecotypes of quinoa. Plants were subjected to 20 mM ${\text{NO}}_{3}^{-}$ or NH_4_^+^ supply per 30 days. Bars are means ± SD (*n* = 6). Two-way ANOVA and Tukey test analysis (*p* < 0.05) were used for to detect differences.

Both Faro and Socaire plants displayed similar A_N_ rates in leaves (normalized by area) under ${\text{NO}}_{3}^{-}$ . Under NH_4_^+^, A_N_ was approximately 50% higher in Socaire than in Faro (*p* < 0.05) (**Figure 7**).

**Figure 8:**
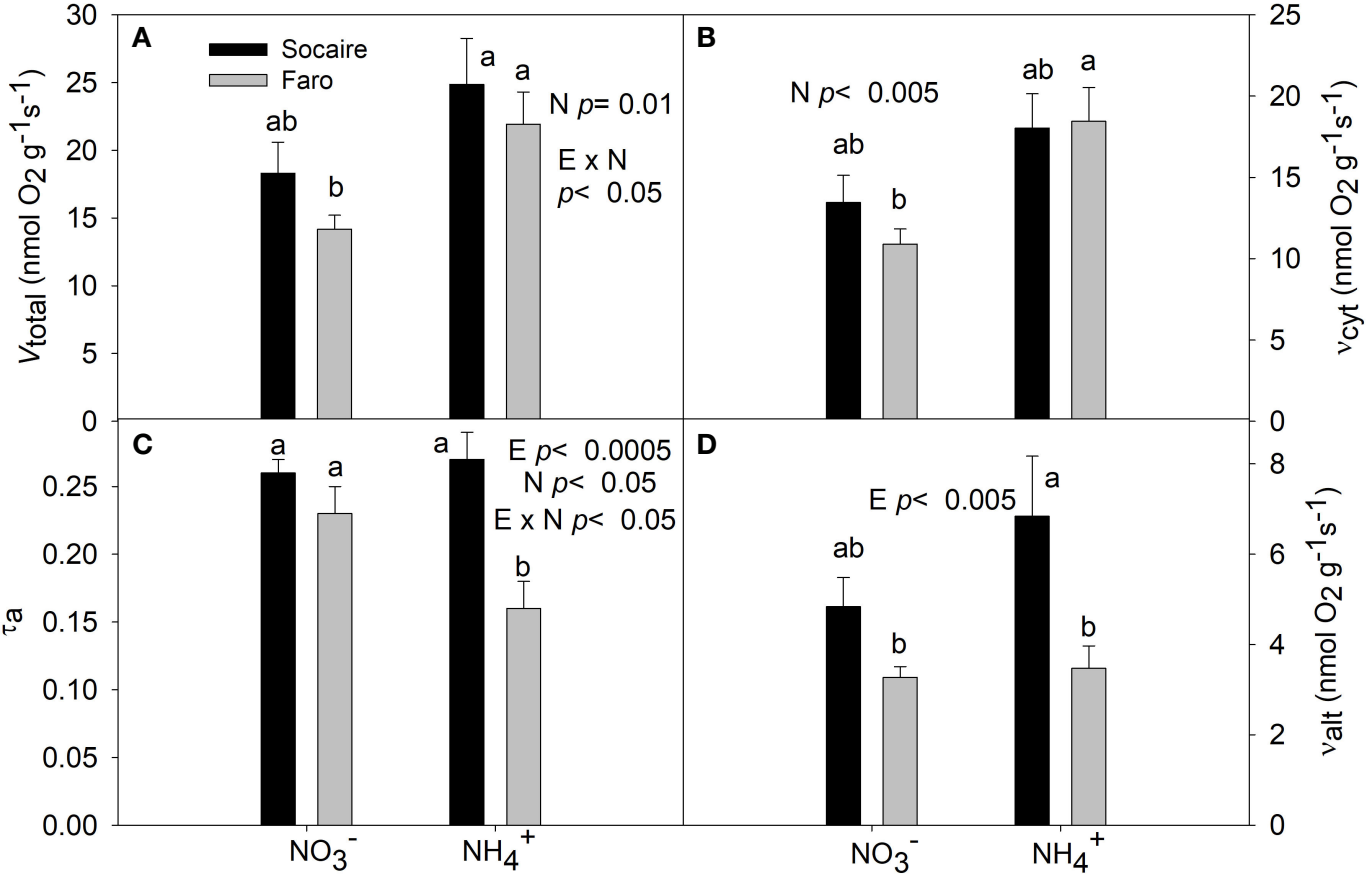
The effect of N sources in total respiration (*V*_total_) **(A)**, cytochrome pathway activity (*v*_cyt_) **(B)**, the electron partitioning to the AOP (τ_a_) **(C)**, and alternative pathway activity (*v*_alt_) **(D)** in leaves of two quinoa ecotypes. Plants were subjected to 20 mM ${\text{NO}}_{3}^{-}$ or NH_4_^+^ supply per 30 days. Values are means ± SE (*n* = 6). Different letters show statistical differences using two-way ANOVA considering ecotypes and source of N as factors. Tukey HSD was used as a *post-hoc* test (*p* < 0.05).

Oxygen consumption and electron partitioning to the COP and AOP under ${\text{NO}}_{3}^{-}$ and NH_4_^+^A significant E × N interaction was observed in total O_2_ uptake (*V*_t_) (*p* < 0.05) and in the electron partitioning to the AOP (τ_a_) (*p* < 0.05). There was a significant reduction (by 30%) of τ_a_ in Faro under NH_4_^+^ compared to ${\text{NO}}_{3}^{-}$ , together with a significant increase (by 80%) in *V*_t_ *via* COP (**Figure 8**). No respiratory changes were observed in Socaire (E × N, *p* < 0.05) (**Figure 8**).

## Discussion

The Chenopodiaceae family has been considered a ${\text{NO}}_{3}^{-}$ specialist and sensitive to NH_4_^+^ (Smirnoff et al., 1984; Britto and Kronzucker, 2002); however, our data showed evidence that the preference for N source depends on the geographical origin of the genotype. When comparing ecotypes under ${\text{NO}}_{3}^{-}$ source, Socaire showed a physiological performance more linked to the N metabolism than Faro, through higher %N, NH_4_^+^, protein, and pigment levels, together with higher root biomass and lower content of MDA (**Figures 1**–**3**, **5**, **6**). However, Socaire showed NH_4_^+^-sensitive growth, even under low NH_4_^+^ concentrations, and under different ${\text{NO}}_{3}^{-}$ :NH_4_^+^ ratios (**Figure 1**; **Supplementary Figures 3**, **4**). Conversely, Faro showed similar biomass accumulation under both ${\text{NO}}_{3}^{-}$ or NH_4_^+^ sources.

Maintaining high C levels is critical to tolerate NH_4_^+^ in soils due to an increased requirement for C skeletons that allow the incorporation of NH_4_^+^ into organic molecules (Wang et al., 2022). Despite the fact that a significant decrease in the C:N ratio was observed in both ecotypes under NH_4_^+^ (**Figure 2**), except for TSS in Faro roots, non-significant changes in non-structural carbohydrates were observed under both N sources (**Figure 4**), which correlated well with the maintenance of photosynthesis in both ecotypes (**Figure 7**). Thus, the biomass restriction in the Socaire ecotype under NH_4_^+^ source appeared to be unrelated to either photosynthesis or carbon storage impairment, which has been observed in other species previously (Ariz et al., 2013; Podgórska et al., 2013; Bittsánszky et al., 2015). Therefore, we searched for other mechanisms that could explain these responses, such as the AOP and COP *in vivo* activities.

Oxygen uptake and the electron partitioning to the AOP were similar in Socaire under both N sources (**Figure 8**). In contrast to Socaire, Faro showed better yielding of respiration by higher *V*_t_ *via* COP (**Figure 8**). A possible explanation for this phenomenon can be based on the occurrence of a “futile ammonium cycling” (Hachiya and Noguchi, 2011), in which the increase of NH_4_^+^ fluxes across the plasma membrane is accompanied by H^+^ extrusion (by the plasma membrane proton-ATPase) to maintain the cytosolic charge balance (Britto and Kronzucker, 2005; Szczerba et al., 2008). This would require large amounts of ATP, helping to explain the increase in O_2_ consumption *via* COP (Kronzucker et al., 2001). In fact, an active H^+^ efflux to avoid cytosolic acidification and to release both proton and acid compounds from inside the cell to the rhizosphere was related to an ammonium-dependent increase of O_2_ uptake in species adapted to acidic soils (Findenegg, 1987; Britto and Kronzucker, 2002; Zhu et al., 2009). Thus, it seems that increases in *V*_t_ and COP activity can contribute to adaptation to acidic soils. On top of this, cell replication and the production of proteins are processes that require the highest quantities of ATP in plants (Liang et al., 2015). In this sense, higher rates of COP in Faro could be a key strategy for the benefit of energy and protein production required for plant development.

Interestingly, Socaire displayed a significantly higher *v*_alt_ than Faro under NH_4_^+^ source. Previous studies suggested a role for AOP during the dissipation of reducing equivalents in cytosol to compensate for the lack of the reductant sink exerted by nitrate reductase under NH_4_^+^ source (Britto et al., 2002; Escobar et al., 2006; Hachiya et al., 2010). Furthermore, the AOP has an important role in dissipating NAD(P)H, under different stress conditions when the COP is impaired, including those prevailing in high mountain habitats, such as cold, low oxygen, and high light intensities (Angert et al., 2012; Jayawardhane et al., 2020). In this sense, the AOP could be more important in Socaire (from Andes mountains) than in Faro (from Lowlands).

In roots, the higher increase of protein content under NH_4_^+^ compared to ${\text{NO}}_{3}^{-}$ in Faro (**Figure 3**) could contribute to avoid toxicity by NH_4_^+^. This is consistent with lower changes in lipid peroxidation under NH_4_^+^ compared to Socaire (**Figure 6**). It has been proposed that the accumulation of NH_4_^+ in^ shoots is more deleterious than in roots (Hachiya et al., 2021); however, the NH_4_^+^ *per se* is not the inductor of ammonium toxicity, but rather an excessive proton production by the incorporation of NH_4_^+^ in Glu to form Gln by glutamine synthase in the chloroplast (Fangmeier et al., 1994; Tobin and Yamaya, 2001; Hofmockel et al., 2010; Hachiya et al., 2021). Thus, the high production of amino acids and proteins in roots could act as a barrier to prevent the transport of NH_4_^+^ to shoots (Hachiya et al., 2021). The increase of proteins in Faro roots under NH_4_^+^ compared to ${\text{NO}}_{3}^{-}$ was related to TTS and starch reductions, supporting the idea that an enhanced NH_4_^+^ assimilation takes place belowground in this ecotype (**Figure 4**). These changes could be related to the donor of C skeletons for the different processes associated with respiration and protein production (glycolysis, tricarboxylic acid cycle, and amino acid production) (De la Peña et al., 2019). Besides amino acids and proteins, pigments are also sinks of NH_4_^+^. Socaire, which showed the highest %N under NH_4_^+^, displayed small changes in protein content under NH_4_^+^ supply compared to ${\text{NO}}_{3}^{-}$ (**Figure 3**), but presented alternative sinks to cope with the excess of NH_4_^+^. Betacyanins (**Figure 5C**) constitute a class of secondary metabolites in Quinoa derived from the amino acids Tyr and DOPA (Sepúlveda-Jiménez et al., 2005). Betacyanins have been related to scavenging ROS under stress conditions but have been inversely related to growth in quinoa (Bascuñán-Godoy et al., 2018). In contrast to Socaire, Faro increased chlorophylls (derived from Glu) under NH_4_^+^ compared to ${\text{NO}}_{3}^{-}$ (**Figures 5A, B**). Chlorophyll has been positively related to both performance of PSII and growth in quinoa (Bascuñán-Godoy et al., 2018). The improvement of betacyanins in Socaire, and the higher level of *v*_alt_, when compared to Faro, may indicate a role of AOP in dissipating energy excess from chloroplasts, helping to maintain homeostasis of metabolism under NH_4_^+^ source. In this sense, a described “trade-off” between the traits of resistance and productivity (Bechtold and Field, 2018) may help to explain the growth reduction observed in Socaire. Conversely, the maintenance of photosynthesis, an enhancement in *V*_t_, and the production of soluble proteins and pigments related to the light collection in Faro suggest the existence of a tight metabolic coordination between chloroplasts and mitochondria. The application of “omics” technologies in future experiments would shed more light on important metabolite pathways for plant performance under NH_4_^+^ source.

## Conclusions

Considering that agroecosystems have the potential to store a vast amount of C, in this work, we highlight the role of soil N sources in the ability to grow and store biomass in two ecotypes of *C. quinoa*. Similar physiological performance was observed in Andean and Lowland ecotypes under ${\text{NO}}_{3}^{-}$ source, but under NH_4_^+^, these showed contrasting C:N relationships that were not related to photosynthesis, but to biomass accumulation and ATP yield of respiration. The enhanced respiration *via* COP in Faro under NH_4_^+^ turned out to be beneficial through the increased energy efficiency of respiration, allowing it to maintain growth, in contrast to Socaire, whose biomass was severely affected. Studies under field conditions and using a wider range of genotypes of each environment are necessary to establish that our findings are a general response. In view of the suggestions about alterations in the N forms available for plants due to climatic variations, increasing our understanding of plant nutrition is relevant, especially in places acutely threatened by climate change, such as the Andean zone.

## Data availability statement

The original contributions presented in the study are included in the article/**Supplementary Material**. Further inquiries can be directed to the corresponding author.

## Author contributions

LB-G, ND-S, EO-G, SF and PC designed the assays and led the writing of the manuscript. MJ conducted all the experimental analysis. JO, CC, EE and CS performed assays and measurements. LB-G, ND-S, MR-C, TP and PC led the supported projects and edited the manuscript. All authors contributed to the article and approved the submitted version.

## Supplementary material

Supplementary Figure 1 Total dry biomass under different N sources and concentrations in two ecotypes of *C. quinoa*. Plants were subjected to different ${\text{NO}}_{3}^{-}$ or ${\text{NH}}_{4}^{+}$ supplies from 0 to 100 mM per 30 days. Values are means ± SE (n = 7). Different letters show statistical differences using three-way ANOVA considering ecotypes and source of N and concentration as factors (Tukey test; *p* < 0.05).

Supplementary Figure 2 C allocation between aboveground and belowground in response to ${\text{NO}}_{3}^{-}$ and ${\text{NH}}_{4}^{+}$ . TSS **(A, D)**, Starch **(B, E)** and NSC **(C, F)** in aboveground and belowground tissues in whole juvenile plants of two ecotypes of *C. quinoa.* Bars are means ± SD (n = 5). Two-way ANOVA of and Tukey test analysis (*p* < 0.05) were used to detect differences.

Supplementary Figure 3 Total dry biomass of Socaire under different ${\text{NH}}_{4}^{+}$ concentrations compared to 20mM ${\text{NO}}_{3}^{-}$ . Plants were grown at different N treatments per 30 days at the same growing conditions described in Material and Methods. Values are means ± SE (n = 5). Different letters show statistical differences using three-way ANOVA considering ecotypes and source of N and concentration as factors (Tukey test; *p* < 0.05).

Supplementary Figure 4 Percent changes in total dry biomass of Socaire (orange) and Faro (blue) under different ${\text{NO}}_{3}^{-}$ : ${\text{NH}}_{4}^{+}$ ratios in two ecotypes of *C. quinoa*. Plants were subjected to 20 mM of N, differing ${\text{NO}}_{3}^{-}$ or ${\text{NH}}_{4}^{+}$ ratios at the same growing conditions described in Material and Methods per 40 days. The studied proportions were: 100:0; 75:25; 50/50; 25:75 and 0:100 of ${\text{NO}}_{3}^{-}$ : ${\text{NH}}_{4}^{+}$ ratios. The 100:0 of ${\text{NO}}_{3}^{-}$ : ${\text{NH}}_{4}^{+}$ ratio was used as Control (100%). Tukey test was used to identify differences regarding 100: 0 ${\text{NO}}_{3}^{-}$ : ${\text{NH}}_{4}^{+}$ (*p* < 0.05). Values are means ± SE (n = 5). For each genotype, ns indicates no significant difference, * indicates *p* < 0.05, ** indicates *p* < 0.01, and *** indicates *p* < 0.001.
