# Supplementary figures and images for: Nitrogen sources differentially affect respiration, growth, and carbon allocation in Andean and Lowland ecotypes of Chenopodium quinoa Willd
Source: Front Plant Sci. 2023 Jun 20;14:1070472. doi: 10.3389/fpls.2023.1070472 (PMC10319013; doi:10.3389/fpls.2023.1070472)

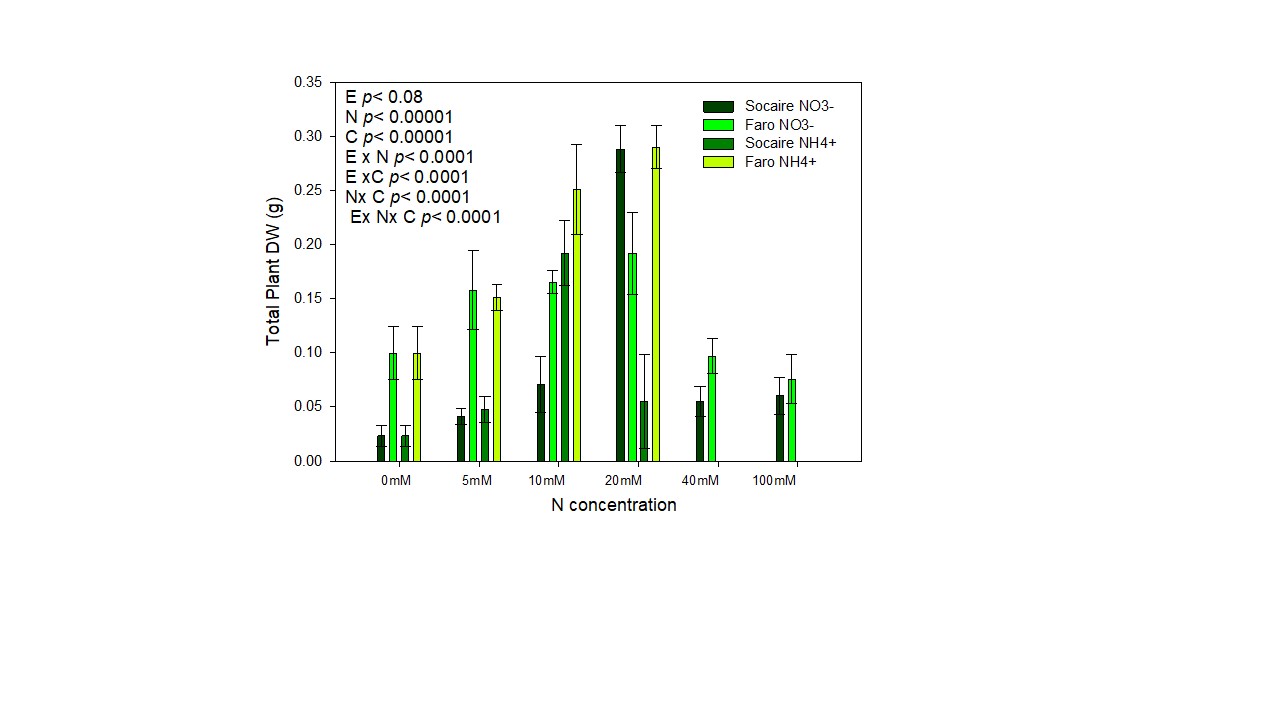

Supplement: Supplementary Figure 1 — Total dry biomass under different N sources and concentrations in two ecotypes of C. quinoa. Plants were subjected to different NO3− or NH4+ supplies from 0 to 100 mM per 30 days. Values are means ± SE (n = 7). Different letters show statistical differences using three-way ANOVA considering ecotypes and source of N and concentration as factors (Tukey test; p < 0.05). [file Image_1.jpeg]

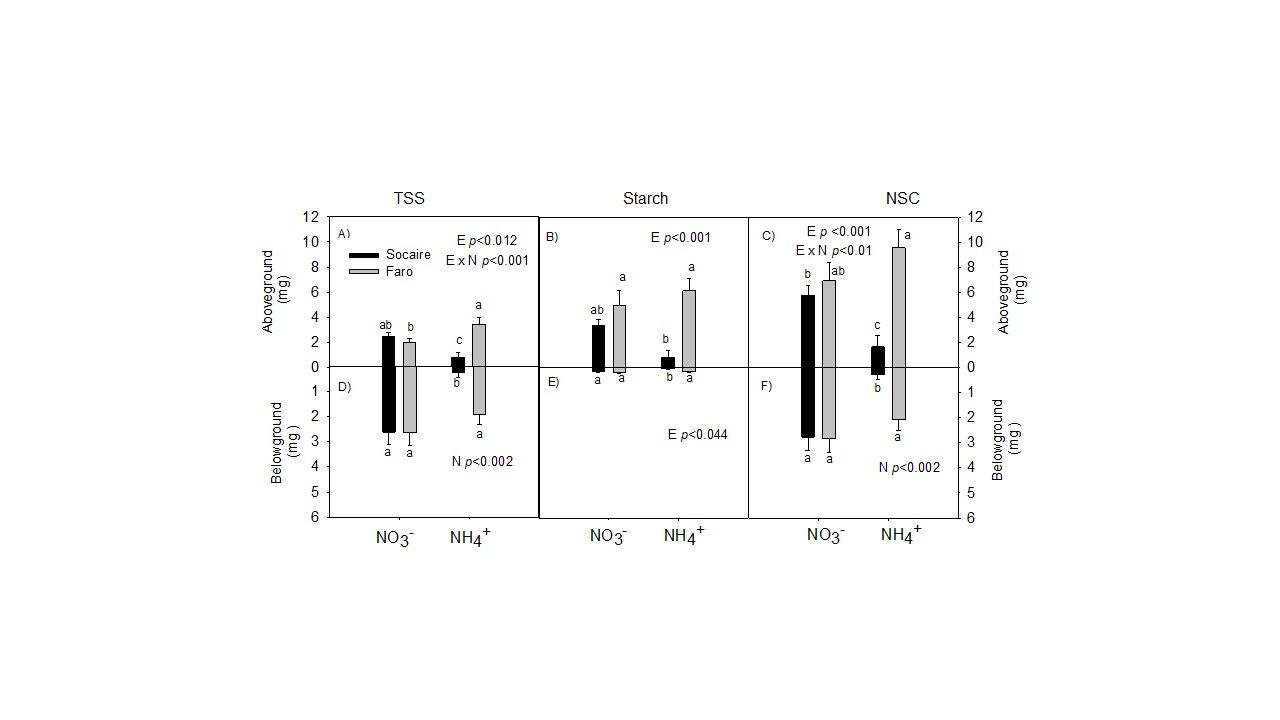

Supplement: Supplementary Figure 2 — C allocation between aboveground and belowground in response to NO3− and NH4+ . TSS (A, D), Starch (B, E) and NSC (C, F) in aboveground and belowground tissues in whole juvenile plants of two ecotypes of C. quinoa. Bars are means ± SD (n = 5). Two-way ANOVA of and Tukey test analysis (p < 0.05) were used to detect differences. [file Image_2.jpeg]

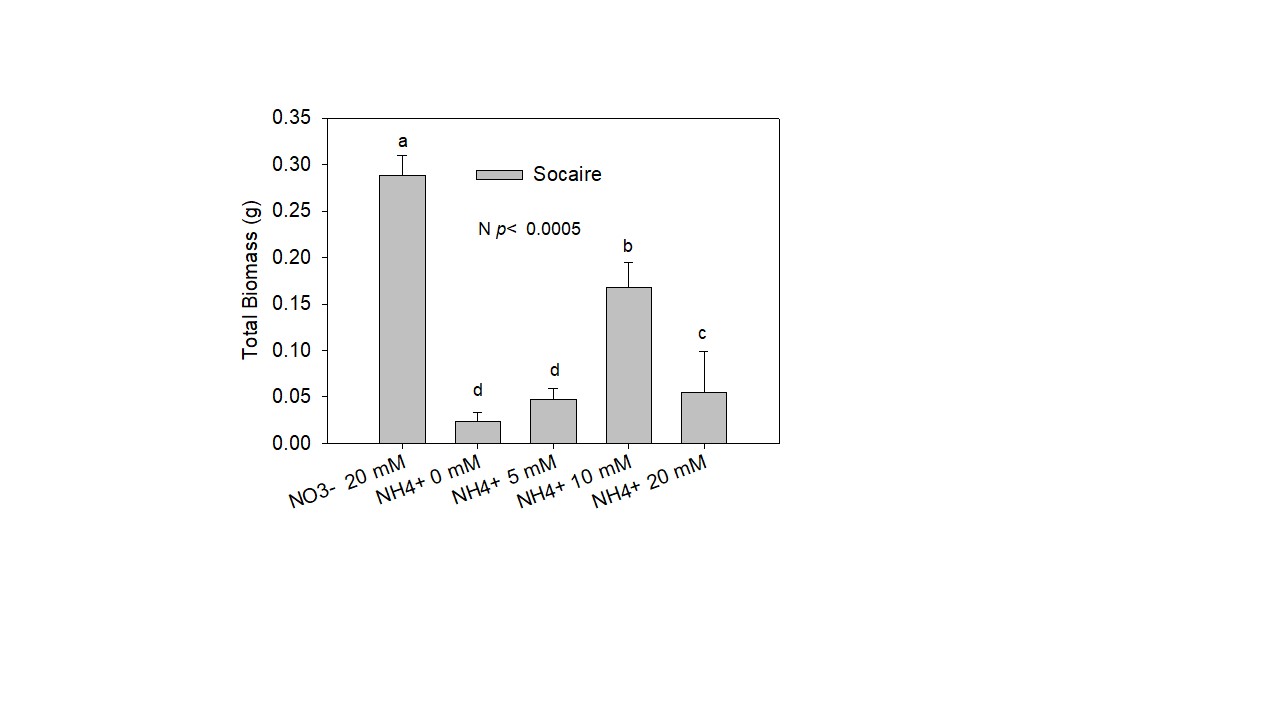

Supplement: Supplementary Figure 3 — Total dry biomass of Socaire under different NH4+ concentrations compared to 20mM NO3− . Plants were grown at different N treatments per 30 days at the same growing conditions described in Material and Methods. Values are means ± SE (n = 5). Different letters show statistical differences using three-way ANOVA considering ecotypes and source of N and concentration as factors (Tukey test; p < 0.05). [file Image_3.jpeg]

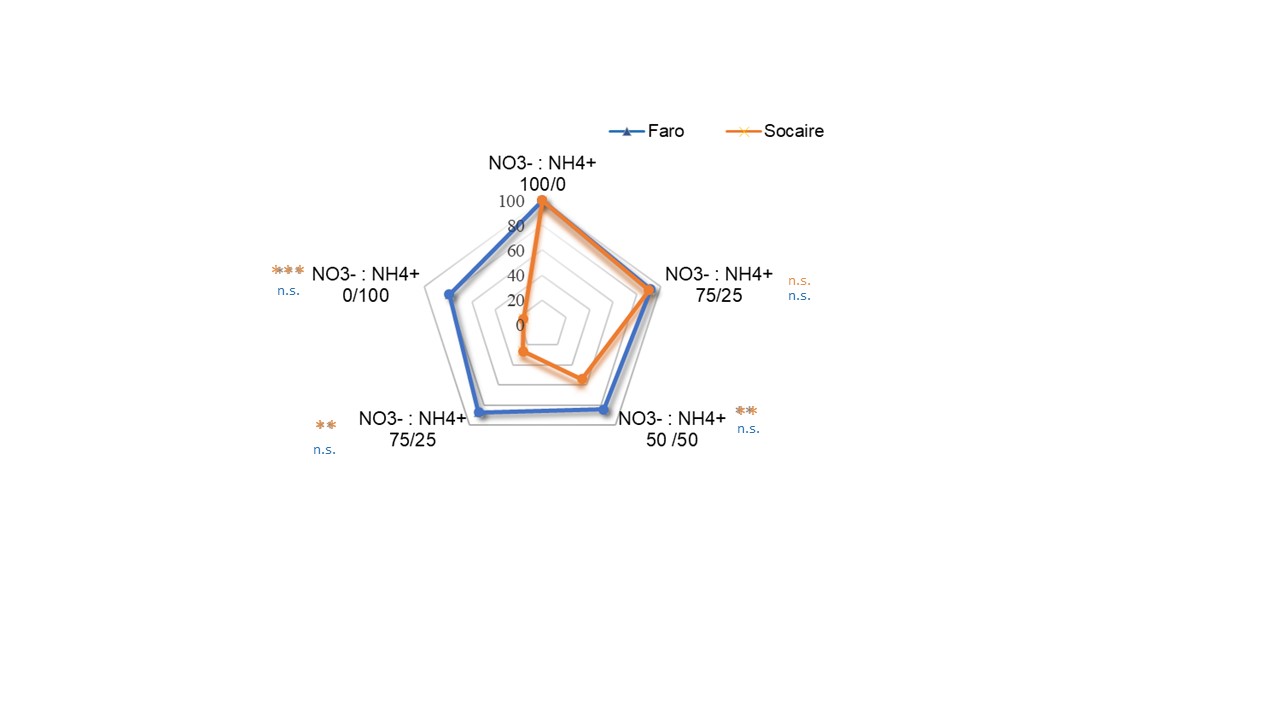

Supplement: Supplementary Figure 4 — Percent changes in total dry biomass of Socaire (orange) and Faro (blue) under different NO3− : NH4+ ratios in two ecotypes of C. quinoa. Plants were subjected to 20 mM of N, differing NO3− or NH4+ ratios at the same growing conditions described in Material and Methods per 40 days. The studied proportions were: 100:0; 75:25; 50/50; 25:75 and 0:100 of NO3− : NH4+ ratios. The 100:0 of NO3− : NH4+ ratio was used as Control (100%). Tukey test was used to identify differences regarding 100: 0 NO3− : NH4+ (p < 0.05). Values are means ± SE (n = 5). For each genotype, ns indicates no significant difference, * indicates p < 0.05, ** indicates p < 0.01, and *** indicates p < 0.001. [file Image_4.jpeg]
